# Short-Term Use of Baclofen Leading to Delirium in a Patient With End Stage Renal Disease Receiving Hemodialysis

**DOI:** 10.7759/cureus.9778

**Published:** 2020-08-16

**Authors:** Muhammad Faisal Riaz, Suhaib A Andrabi, Anya Weerasinghe, Maryam Handoo, Sudhanshu Jain

**Affiliations:** 1 Internal Medicine, Harlem Hospital Center, New York, USA; 2 Nephrology, Harlem Hospital Center, New York, USA

**Keywords:** baclofen, muscle relaxant, end stage renal disease, hemodialysis, delirium

## Abstract

Baclofen, a muscle relaxant is primarily excreted by the kidneys. We present the case of a 60-year-old male who developed acute delirium, visual hallucinations, and behavioral changes after he took a friend’s prescription of baclofen for pain relief. There was immediate improvement after dialysis, and multiple sessions of hemodialysis led to complete resolution of symptoms. A high suspicion for pharmacological causes of delirium is needed in the end stage renal disease (ESRD). Baclofen is known but under recognized cause of acute delirium in ESRD patients. We discuss the pharmacokinetics of baclofen. We suggest reducing dose or preferably avoiding use of baclofen in ESRD patients, especially the elderly.

## Introduction

Baclofen is a known but uncommon cause of altered mental status. It is a gamma aminobutyric acid (GABA) agonist and acts by inhibiting polysynaptic and monosynaptic reflexes at spinal cord [1]. It is mainly used as a muscle relaxant and to treat muscle spasticity. Baclofen can lead to multiple side effects including cardiovascular system (CVS) and central nervous system (CNS) among other systems. Neurological side effects include delirium, seizures, altered mental status, and coma [1]. It can also lead to sleepiness, weakness, dizziness, and rhabdomyolysis. Daily dose above 200 mg is strongly associated with toxicity and hemodialysis patients are at high risk. Our patient reported after just taking three tablets of baclofen with a total dose of 30 mg. Response to hemodialysis was documented and led to slow recovery from toxicity of baclofen [2-3].

## Case presentation

**Figure 1:**
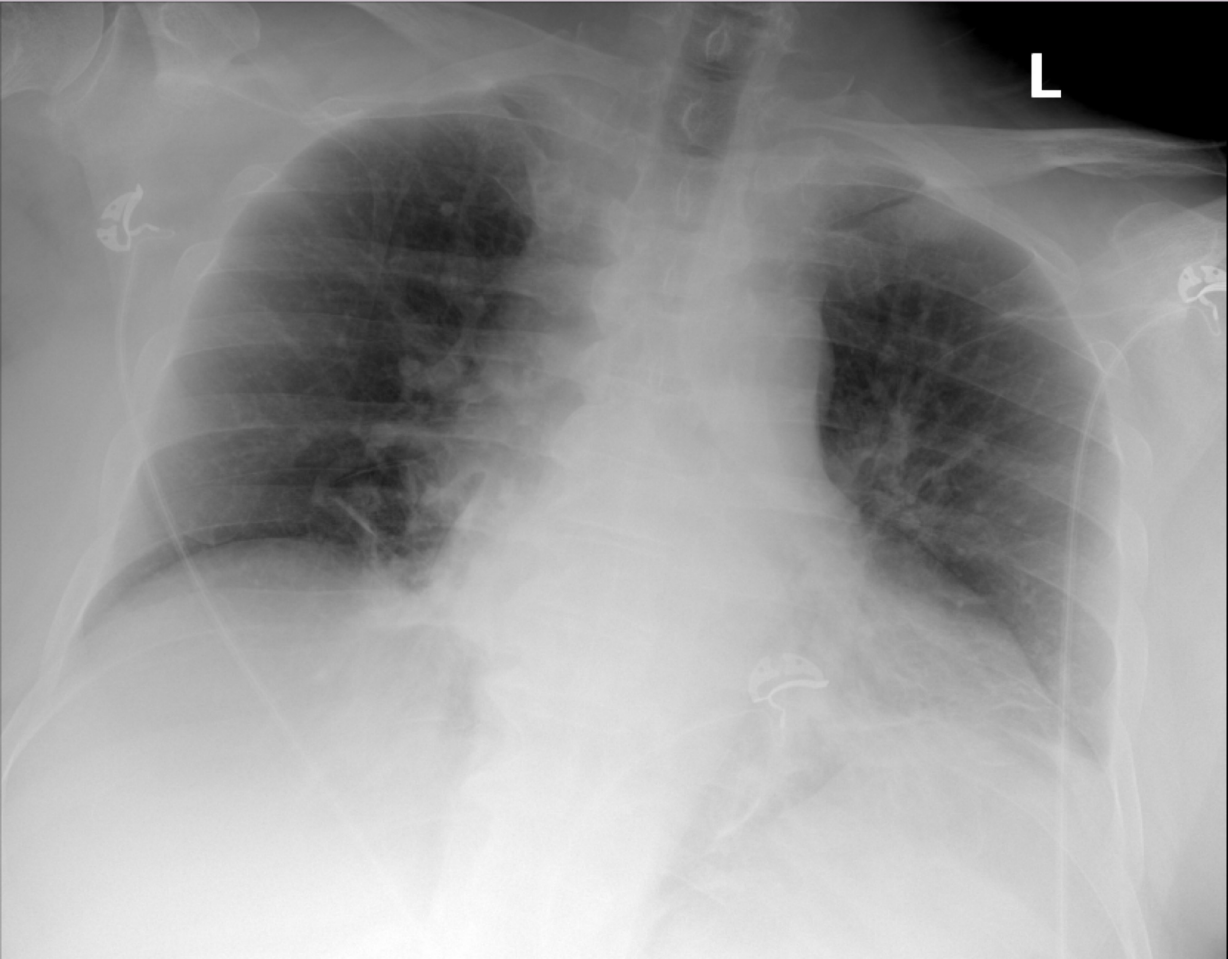
CXR showing left lung atelactasis/pleural effusion. CXR, chest X-ray

A 60-year-old male was brought in by emergency medical services (EMS) for altered mental status. He was in usual state of health prior to being found lying in his bathroom, according to relatives. According to his daughter he was fine until last seen well few hours ago. He lived alone and had no home health aide. He had a past medical history (PMH) of ESRD on hemodialysis three days a week. He also had PMH of hypertension (HTN), type 2 diabetes mellitus, hyperlipidemia, coronary artery disease s/p cardiac catheterization in 2015, heart failure with reduced ejection fraction, and deep vein thrombosis (DVT) on apixaban. According to his daughter he appeared to have taken three tablets of baclofen. On arrival to ER he was confused and obtunded. His pulse was 84/min, blood pressure (BP) 209/78 mmHg, respiratory rate (RR) 16/min, temperature 97.4 degree Farenheit, and he was saturating 96% on 2L of nasal cannula. He was drowsy and unable to follow commands. He had no neck rigidity and Kernig’s sign was negative. His pupils were equal and reactive to light and he had no nystagmus. He had no crackles on chest exam and there was no pedal edema. Laboratory workup and findings are listed in the table below (Table 1). CT brain did not show evidence of mass, hydrocephalus, acute intracranial hemorrhage, abnormal extra-axial fluid collection, or obvious cortical infarct in the brain. Stroke and posterior reversible encephalopathy syndrome were ruled out. Electrocardiogram (EKG) showed normal sinus rhythm. Chest X-ray (CXR) showed left basilar atelectasis/consolidation with or without pleural effusion (Figure 1). Initially he was admitted to ICU for management of suspected sepsis and meningitis was also one of the differential diagnosis. He was started on broad-spectrum antibiotics. Nephrology team was consulted and he received three hours and 30 minutes of hemodialysis. After the first session of dialysis he was more arousable and alert, but appeared to be agitated and had visual hallucinations. Baclofen level sent after first hemodialysis session was 0.10 mcg/mL (upper limit 0.20 mcg/mL). His antibiotics were stopped as there was no focus of infection and he was not febrile. He was stepped down to medical floors. Psychiatry service was also consulted and agitation was managed with haldol. They felt that visual hallucinations suggested an organic cause of his psychiatric symptoms. We concurred with psychiatry as to possible metabolic/drug induced cause of neurological symptoms. The fact that there was improvement after one session of dialysis suggested this as well. Redistribution could account for recurrence of delirium. He underwent further sessions of hemodialysis with improvement in mentation. He was alert, awake and oriented to time, place, and person (AOAX3). He recalled that he got baclofen from his friend. He never had taken baclofen before. He recalled that he was drowsy and was transferred to the hospital the same day. He was discharged home with follow up with his own dialysis center for dialysis three days a week. He followed up with his primary care physician (PCP) and was getting regular dialysis and remained symptom free. 

**Table 1:** Laboratory findings and results. WBC, white blood cell; Na, sodium; K, potassium; BUN, blood urea nitrogen; Cl, chloride; CO2, bicarbonate; Day 0, predialysis; Day 1, 3, 4, postdialysis

Work up	Day 0 (admission)	Day 1	Day 3	Day 4	Normal ref. range
WBC	13.61	14.98	10.19	10.98	4.80-10.8 X 10^3^/mcl
Neutrophil	80.3	92.1	67.7	63.9	44%-70%
Platelets	256	235	286	279	150-400 × 10^9^/L
Na	132	139	137	139	136-145 mmol/L
K	5.3	4.1	3.6	4.3	3.5-5.1 mmol/L
Cl	91	97	93	98	98.107 mmol/L
CO_2_	19	22	23	23	22-29 mmol/L
BUN	78	28	27	21	7-18 mg/dL
Creatinine	6.5	2.5	3.3	3.8	0.7-1.2 mg/dL
Alcohol	<10	-	-	-	<10 mg/dL
Salicylate	<0.3	-	-	-	3.0-10 µg/L
Lithium	<0.05	-	-	-	0.60-1.2 nm/L
Acetaminophen	<5	-	-	-	10.0-30.0 µg/mL
Carbamazepine	<2.0	-	-	-	4-12 µg/ml
Phenytoin	1.2	-	-	-	10.0-20.0 µg/mL
Valproic acid	<3	-	-	-	50-100 µg/mL
Phenobarbitol	<2.4	-	-	-	10-300 µg/mL
Baclofen level		0.10			Upper limit 20.0 µg/mL

## Discussion

Baclofen, a GABA agonist is highly lipophilic. It is considered as an active agonist for GABAb receptors. It also acts as an inhibitor in the dopaminergic pathway. It induces inhibition at presynaptic motor neurons. It is used to treat muscle spasticity due to spinal diseases, multiple sclerosis, and hiccups. It is absorbed primarily by the gastrointestinal (GI) tract. It is primarily excreted by kidneys (85%) and also cleared by liver. At 72 h 75% of the administered dose is recovered unchanged in urine and 5% metabolites in healthy objects [1]. Its half-life is 4.5-6.8 h and it increases in patients with renal failure. It can also cross blood-brain barrier. Oral baclofen toxicity is rare but can occur in patients with impaired renal function [2]. Poor awareness of renal dose adjustment is responsible for the toxic effects.

Baclofen toxicity includes but is not limited to dizziness, nausea, vomiting, respiratory depression, altered mental status, delirium, ataxia, dystonia, drowsiness, lethargy, and severe comatose state [1, 3]. Altered mentation is a major manifestation in renal impairment. Symptoms develop soon after the start of baclofen intake. Toxicity can occur even after a single dose in patients with impaired renal functions and ESRD patients on hemodialysis. Baclofen has small molecular weight, low volume of distribution, and relatively low protein binding, and therefore dialyzable. Although percentage of clearance varies with each person, it can be 79% cleared in one dialysis session. Hemodialysis can reduce half-life of baclofen to 15-2h and removes baclofen as effectively as normal kidneys would do [2-3].

Hemodialysis is effective in removal and is the preferred treatment modality and is the most effective treatment [4-5]. In our patient dramatic response was seen with one session of dialysis [4]. As reported in literature, rebound symptoms occurred after hemodialysis and improved after multiple dialysis sessions. Treatment of baclofen overdose is supportive and there is no reversal for baclofen at present.

## Conclusions

Baclofen is used as a muscle relaxant and prescribed for muscle spasms and rigidity. It is associated with significant side effects in dialysis patients including confusion, altered mental status, and dizziness. It can lead to quick toxic effects in patients with impaired renal functions as compared to patients who have adequate renal functions. It is effectively cleared with hemodialysis. In conclusion it should be used with caution in ESRD patients and alternate medications should be selected. It should be avoided in patients with glomerula filtration rate (GFR) less than 30 and on renal replacement therapy. Assessment risk versus benefit is required when considering the use of baclofen for patients with renal impairment, chronic kidney disease, or those on renal replacement therapy. There is a need for greater awareness and education on the possible dangers of prescribing baclofen, prompt diagnosis, and management of toxicity.
